# The Impact of Varying Lactose-to-Maltodextrin Ratios on the Physicochemical and Structural Characteristics of Pasteurized and Concentrated Skim and Whole Milk–Tea Blends

**DOI:** 10.3390/foods13183016

**Published:** 2024-09-23

**Authors:** Dilema Wijegunawardhana, Isuru Wijesekara, Rumesh Liyanage, Tuyen Truong, Mayumi Silva, Jayani Chandrapala

**Affiliations:** 1 School of Science, STEM College, RMIT University, Bundoora, VIC 3083, Australiamayumi.silva@rmit.edu.au (M.S.); 2 Department of Biosystems Technology, Faculty of Technology, University of Sri Jayewardenepura, Dampe-Pitipana Road, Homagama 10200, Sri Lanka; 3 Department of Food Science and Technology, Faculty of Applied Sciences, University of Sri Jayewardenepura, Gangodawila, Nugegoda 10250, Sri Lanka; 4 School of Science, Engineering & Technology, RMIT University, Ho Chi Minh City 700000, Vietnam

**Keywords:** milk–tea, lactose, maltodextrin, protein interactions, milk fat

## Abstract

This study investigates the impact of substituting lactose with maltodextrin in milk–tea formulations to enhance their physicochemical and structural properties. Various lactose-to-maltodextrin ratios (100:0, 90:10, 85:15, 80:20, 75:25) were evaluated in both post-pasteurized and concentrated skim milk–tea (SM-T) and whole milk–tea (WM-T) formulations. Concentration significantly improved the zeta potential, pH, and browning index in both SM-T and WM-T compared to pasteurization. L:M ratios of 90:10 and 75:25 in WM-T and 90:10 and 80:20 in SM-T showed higher phenolic preservation after concentration due to structural changes resulting from the addition of maltodextrin and water removal during prolonged heating. The preservation effect of phenolic components in both WM-T and SM-T is governed by many mechanisms including pH stabilization, zeta potential modulation, protein interactions, complex formation, and encapsulation effects. Therefore, optimizing milk–tea stability and phenolic preservation through L:M ratio adjustments provides a promising approach for enhancing milk–tea properties.

## 1. Introduction 

Milk–tea, a valued and nutritious beverage, includes a diverse array of constituents. It is available in two primary forms: liquid and powdered. Liquid milk–tea blends utilize liquid milk with tea infusion, while powdered milk–tea utilizes tea powder and milk powder. These components can be either dry-mixed or employed as raw materials (wet-mixed) [1,2]. The quality of the final product in the dry-mixing method depends upon the ingredient quality, given that the powder undergoes no additional heat treatments. Conversely, wet-mixing involves adding tea infusion to milk, followed by pre-heat treatment, concentration, and dehydration to produce powdered milk–tea blends. This method ensures uniform nutrient distribution and reduces dependency on base ingredients for microbiological quality [3,4,5]. 

Lactose, constituting 4–5% of milk’s composition, serves as its primary carbohydrate [6]. However, in milk-containing formulas, lactose is sometimes partially replaced by other carbohydrates like maltodextrin to enhance the techno-functional properties. Regulations allow powdered milk-containing formulas to be fortified with up to 30% maltodextrin from the total carbohydrate [7]. Maltodextrin, an approved carbohydrate additive in such formulas, provides valuable energy, and this is especially true for digestible maltodextrin with a Dextrose Equivalent (DE) below 20, as the resulting glucose from its digestion is easily absorbed in the small intestine for metabolic processes [8]. Additionally, maltodextrin offers several technological advantages, including enhanced bulk density, solubility, viscosity, volume, oral sensation, and stability [9,10]. 

Thermal processing methods like pasteurization, concentration, and spray drying exert strong effects on the molecular structures of proteins, polyphenols, fats, and carbohydrates [11,12,13]. These processes induce structural alterations within the internal composition of molecules, leading to molecular grafting, conjugation, or polymerization. Consequently, the reconfigured molecules can significantly modify the physical and chemical properties of the product, thereby influencing the expected characteristics of the final product [14]. Native whey proteins play a major role in these interactions due to their distinctive structural arrangements [15,16,17]. Upon heating, proteins undergo denaturation and unfold from their globular structure, resulting in protein–protein interactions and aggregation. However, lactose and fat prevent whey protein denaturation and unwanted aggregation by enhancing protein stability [18]. While lactose enhances protein stability, its higher reactivity compared to maltodextrin can impact the Maillard reaction and the overall quality of the final formulation [19,20]. 

Milk–tea is a complex mixture consisting of milk proteins, fat, polyphenols, minerals, and lactose. Maltodextrin is commonly added to milk–tea powder to improve yield and solubility, but little is known about how different lactose-to-maltodextrin ratios impact milk–tea blends. Recent studies have explored how lactose and maltodextrin influence infant formulas [7,20,21,22]. Maltodextrin and lactose can interact with proteins and fats during processing, affecting the quality of the final product [23,24,25]. Understanding these effects, along with the interactions of polyphenols, fat, and milk proteins in milk–tea blends, could enhance the development of instant milk–tea formulas. Thus, this study aims to explore the influence of the lactose-to-maltodextrin (L:M) ratio on the physical and structural properties of both liquid and concentrated whole and skimmed milk–tea formulations.

## 2. Materials and Methods

### 2.1. Materials

Orange Pekoe tea which was used as the tea source was provided by Dilmah Ceylon Tea Company (Paliyagoda, Sri Lanka). Whole milk powder (containing 41.59% (*w*/*w*) lactose, 25.28% (*w*/*w*) protein, 27.14% (*w*/*w*) fat, and 5.99% (*w*/*w*) minerals) and skim milk powder (containing 54.2% (*w*/*w*) lactose, 35.4% (*w*/*w*) proteins, 1.3% (*w*/*w*) fat, and 9.11% (*w*/*w*) minerals) were purchased from Fonterra Ltd. (Auckland, New Zealand). Maltodextrin (rice-based; 10–14 Dextrose Equivalent, 100% (*w*/*w*)) and lactose anhydrous (containing lactose 98% (*w*/*w*)) were purchased from Roquette India Private Limited (Mumbai, India) and Research-Lab Fine Chem Industries (Maharashtra, India), respectively. Pre-cast gels (4–20%) were obtained from Bio-Rad. All chemicals used were of analytical grade and were obtained from Sigma Aldrich Pty Ltd. (Castle Hill, NSW, Australia) or Bio-Rad Laboratories Pty Ltd. (Gladesville, NSW, Australia). MilliQ water was used in all experiments.

### 2.2. Methods

#### 2.2.1. Preparation of Black Tea Infusions 

The black tea infusions were prepared as described by Chen et al., (2020) [26], with some modifications to the steeping time and temperature according to Irakli et al., (2018) [27]. A weight of 10 g of tea leaves was steeped in 500 mL of MilliQ water (2% *w*/*v*) at 87.5–99.8 °C for 10 min to extract a polyphenol-rich infusion. After the extraction of tea infusion, coarse filtering was performed, followed by fine filtering through a 1 mm sieve. The extract was then cooled to 50 °C, and an aliquot with a volume of approximately 200 mL was taken for formulation.

#### 2.2.2. Formulation of Milk–Tea Blend

Skim milk and whole milk samples were prepared with varying lactose-to-maltodextrin (L:M) ratios (as summarized in Table S1), using the method outlined by Masum et al., (2019) [7]. Five different L:M ratios (100:0, 90:10, 85:15, 80:20, 75:25) were created for both skim and whole milk. First, 200 mL skim milk and whole milk solutions were prepared by dissolving 90 g of skim and whole milk powder in 110 mL of MilliQ water heated to 50 °C. The appropriate amount of lactose was initially added to the skim and whole milk and dissolved at 50 °C for 10 min, followed by the dissolution of maltodextrin. On a dry basis, the carbohydrate content of 52.8% (*w*/*w*), protein content of 22.8% (*w*/*w*), and fat content of 24.4% (*w*/*w*) were maintained for whole milk formulas, while for skim milk formulas, the target composition was maintained at a carbohydrate content of 68.1% (*w*/*w*) and protein content of 31.9% (*w*/*w*). The total solid (TS) content for all milk samples was maintained at 20% (*w*/*w*) using MilliQ water. 

Then, milk was added to the tea infusion at 50 °C. The mixture was then strained through a 1 mm sieve to remove any clumps or sediments. The pH of all dispersants was adjusted to 6.8 using food-grade 1 M sodium bicarbonate (NaHCO_3_) at 25 ± 2 °C. Then, samples were heated in a water bath at 62.8–65.6 °C for 30 min for pasteurization [28]. The pasteurized formulations were homogenized using a hand blender (GHB5468, GEEPAS, Jabel Ali, Dubai, United Arab Emirates) and then stored at 4 °C overnight to allow for further hydration until they reached equilibrium. The next day, the prepared samples were divided into two portions. One portion was used to analyze the changes induced by pasteurization, while the other portion was concentrated. Concentration was achieved by gently and continuously agitating the formulations at 62–65 °C using direct heating until a solid content of 50–60% was obtained. The resulting formulations were then analyzed to assess the changes induced by the concentration process.

Milk–tea formulations with the original milk composition served as the control samples for both whole milk–tea and skim milk–tea.

### 2.3. Physicochemical Characterization of Liquid and Concentrated Milk–Tea Formulas

#### 2.3.1. pH

The pH of both liquid and concentrated samples was tested using a digital pH meter (SevenCompact pH/Ion, Mettler Toledo, Columbus, OH, USA). The pH probe was calibrated at 25 °C using standard buffer solutions at pH 4.0, 7.0, and 10.0 before each measurement.

#### 2.3.2. Color

Color analyses were conducted using a digital chroma meter (CR-400, Konica Minolta Sensing Americas Inc, Ramsey, NJ, USA), and L*, a*, and b* values were measured following the calibration of the equipment. The L* value represents lightness, the a* value indicates the intensity of red or green color, and the b* value reflects the intensity of yellow or blue color [29]. The purity of the brown color was measured by the browning index (BI) as described by de Oliveira et al., (2021) [30].

$$Browning\;index=\frac{100\;(x-0.31)}{0.172}$$

where *x* is

$$x=\frac{\left(a^{*}+1.75L^{*}\right)}{\left(5.645L^{*}+a^{*}-3.012b^{*}\right)}$$

#### 2.3.3. Total Phenolic Content (TPC)

The changes in the TPC of fortified milk–tea formulas were determined using spectrophotometry, with gallic acid as the standard (R^2^ = 0.9967), as described by Uduwana et al. (2023) [31] with some modifications. Briefly, working solutions were prepared by dissolving 1.5 mL and 0.5 mL of pasteurized and concentrated milk–tea solutions, respectively, in 100 mL volumetric flasks and diluting them with MilliQ water. Then, 1 mL of each diluted milk–tea sample was mixed with 5 mL of 10% (*v*/*v*) Folin–Ciocalteu solution and 4 mL of 7.5% (*w*/*v*) sodium carbonate solution. The mixture was left in the dark at room temperature for two hours, and absorbance was measured at 765 nm using a UV spectrophotometer (SHIMADZU, UV mini-1240, Guelph, ON, Canada) against MilliQ water. The TPC was expressed as gallic acid equivalent (GAE) in µg/mL of milk–tea. 

#### 2.3.4. Zeta Potential and Z-Average Particle Size 

The surface charge of the liquid and concentrated milk–tea particles was measured using a Zeta Potential Analyzer (Brookhaven Instruments v.3.6, Nashua, NH, USA), and the Z-average particle size was measured using a Zetasizer (Zetasizer Ver.7.12, Malvern Instruments Ltd., Malvern, UK) at 25 °C. The samples were diluted 100 times [32,33,34]. The refractive index for particle size of SM-T and WM-T was considered as 1.50 and 1.46. The absorption value was set as 0.001 during measurements [35].

### 2.4. Structural Characterization of Liquid and Concentrated Milk–Tea Formulas 

#### 2.4.1. Fourier Transform Infrared Spectroscopy (FTIR)

Secondary structural changes of proteins in concentrated SM-T and WM-T were monitored using an FTIR instrument (ThermoFisher Scientific Inc., Waltham, MA, USA). The measurements were taken with an average of 16 scans at 4 cm^−1^ resolution within the 400–4000 cm^−1^ range after background subtraction. The resulting spectra were analyzed using Origin software (OriginPro 2024, OriginLab Corp, Northampton, MA, USA). Second derivative spectra of WM-T and SM-T concentrate were used in the region of 1600–1700 cm^−1^, known as the amide I region [36]. The band assignment was performed based on previous studies [37,38] where β-sheets (1610–1642 cm^−1^ and 1688–1697 cm^−1^), random coils (1644–1648 cm^−1^), α-helix structures (1651–1653 cm^−1^), large loops (1656–1658 cm^−1^), and β-turns (1667–1684 cm^−1^) were assigned.

#### 2.4.2. Sodium Dodecyl Sulfate Polyacrylamide Gel Electrophoresis (SDS PAGE)

Individual native whey and casein bands were confirmed using non-reducing SDS PAGE, following the method described by Mejares et al., (2023) [39]. The concentrated SM-T and WM-T were diluted at a 1:1 ratio of sample and distilled water before ultracentrifugation. The clear supernatant was carefully separated using a syringe after centrifugation. First, 10 µL of extracted soluble protein was mixed with Laemmli sample buffer and vortexed. The samples were then heated at 70 °C for 10 min. Next, 10 µL of prepared samples and protein standard (BioRad Precision Plus Protein Standard, South Granville, NSW, Australia) were injected into pre-cast 4–20% Mini-PROTEAN TGX (BioRad, South Granville, NSW, Australia) gels inserted into a Mini PROTEAN Tetra vertical electrophoresis cell (BioRad, South Granville, NSW, Australia). The gels were run for 60 min at 150 V and then rinsed in Mili-Q water for 15 min before staining with Bio-Safe Coomassie G-250 stain (BioRad, South Granville, NSW, Australia). The gels were then left in the staining solution overnight with continuous agitation, followed by rinsing with Mili-Q water. Protein bands were visualized using the ChemiDoc MP Imaging System (Bio-Rad, Hercules, CA, USA), and their intensities were quantified using ImageJ software (version 8) to determine the quantity of individual proteins; relative band intensity was calculated using the following equation.

$$\%\;of\;individual\;protein\;fraction=\frac{Intensity\;of\;Specific\;Protein\;Band}{Total\;intensity\;of\;all\;individual\;bands}\times 100$$

#### 2.4.3. Polarizing Microscopy 

The liquid and concentrated milk–tea samples were analyzed using an Olympus BX53 polarizing microscope (Nikon Instruments, Melville, NY, USA) with polarized light, following the method outlined by Maher et al., (2015) [40]. Digital images were captured using a Jenoptik Imagic camera (Nikon Instruments, Melville, NY, USA), with 20× and 40× objectives used in the optical path. Crystalline regions were observed as bright areas in the micrographs.

### 2.5. Statistical Analysis

The data were analyzed using IBM SPSS software (version 26). All measurements were performed in triplicate, and average values were compared. The data were subjected to ANOVA to determine the mean differences and homogeneous subsets. The significance level was set at *p* < 0.05, and Tukey’s HSD test was used for post hoc multiple comparisons of the means. Multiple Linear Regression was used, and Pearson correlation coefficient (*r*) was computed to assess the relationships. 

## 3. Results and Discussion 

### 3.1. Changes in the Physicochemical Properties of Liquid and Concentrated Milk–Tea Affected by Varying L:M Ratios

#### 3.1.1. pH and Zeta

**Figure 1:**
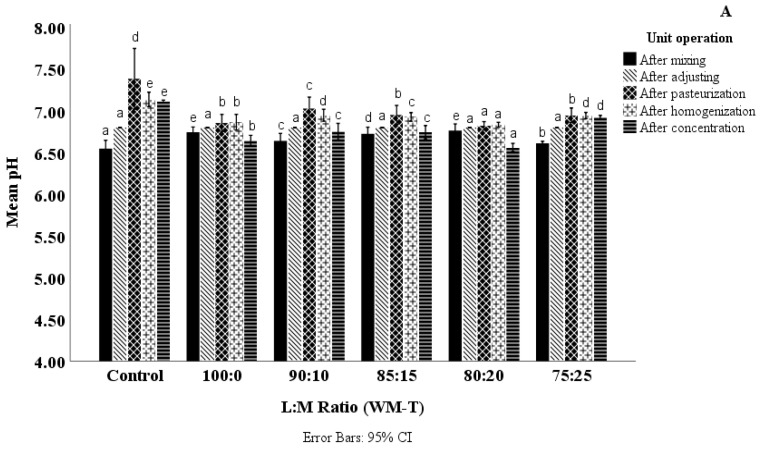

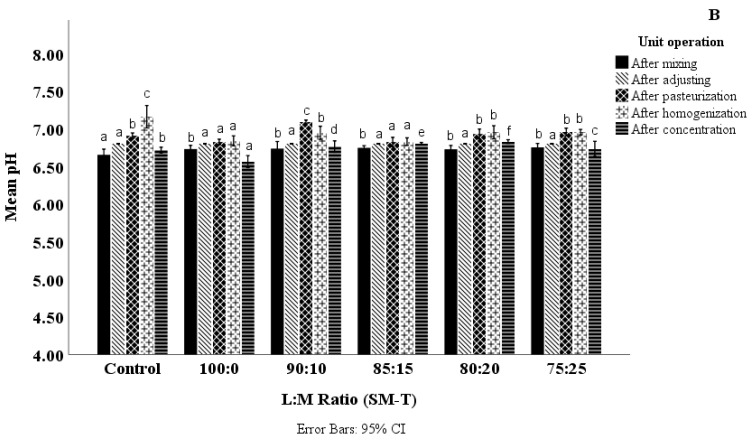
pH changes of (**A**) WM-T and (**B**) SM-T with varying L:M ratios during mixing, adjusting pH, pasteurization, homogenization, and concentration (Refer to Table S2); Lowercase superscripts within each processing stage (mixing, pasteurization, homogenizing, and concentration) for the same milk–tea formulation (WM-T and SM-T) indicate significant differences (*p* < 0.05).

Figure 1A,B illustrate the pH variations observed for different L:M ratios of WM-T (A) and SM-T (B) throughout the different processing stages of mixing, pasteurization, homogenization, and evaporation (refer to Table S1). Prior to pasteurization, WM-T exhibited a slightly lower pH (6.55 ± 0.04) compared to SM-T (6.65 ± 0.03). The presence of fat influences the initial acidity levels, potentially due to the higher number of free carboxylic (–COOH) groups found in fat molecules. These free -COOH groups can dissociate, releasing H^+^ ions into the solution, thereby increasing its acidity [41]. Both WM-T and SM-T zeta potential values were higher at −30 mV compared to the zeta potential values reported by Youravong et al., (2002) [42] for milk at pH 6.2 to 7.7, which ranged from −16.1 to −20.1 mV. The elevated zeta potential values of both WM-T and SM-T are attributed to tea polyphenols in the formulations, which contribute to the negative charges on the protein-rich interfacial layers due to the presence of anionic groups in some of the molecules within the tea polyphenols.

The addition of lactose and maltodextrin increased the pH of both WM-T and SM-T samples. This is likely due to the dilution effect created by the additions, where milk’s acidic components are diluted and the concentration of H^+^ ions is reduced, as stated by Salaun et al., (2005) [43]. Pasteurization led to an increase in pH irrespective of the L:M ratio. Upon pasteurization, the pH of WM-T increased to 7.38 ± 0.15, compared to 6.91 ± 0.02 for SM-T. This pH increase upon heating contrasts with whole milk and skim milk alone where a decrease in pH or almost no change in pH was detected by various studies [44,45,46]. This is due to various reasons. One reason is the interaction between polyphenols and milk proteins, leading to the sequestration of H^+^ ions and reducing the overall acidity. Furthermore, the interaction of milk proteins with polyphenols can alter the buffering equilibrium [47]. Additionally, the ionization state of polyphenols can change with these interactions, causing the release of some H^+^ ions. Polyphenols, which are excellent sources of antioxidant properties, can influence the redox balance in milk, affecting the dissociation of certain acidic components and leading to an increase in pH [48,49]. The WM-T increase was much more pronounced compared to SM-T due to polyphenol binding with free fatty acids, which reduces the number of free acidic groups in the system, contributing to a higher pH. Additionally, it can be attributed to a reduction in carboxyl groups of fat resulting from cross-linking reactions between maltodextrin and fat, involving hydrogen bonding between the hydroxyl group of maltodextrins and the carboxyl group of fat [50,51]. Zeta potential values aligned well with pH data, where an increasing concentration of maltodextrin tended to reduce the zeta potential for both SM-T and WM-T. 

Concentration decreased pH but increased surface charge irrespective of whether fat was present (*r*^2^ = −0.091, *p* < 0.05) or not (*r*^2^ = 0.084, *p* < 0.05) (Table 1). Concentration affects a significant decrease in moisture content, leading to an increase in the concentration of solutes such as proteins, fats, and sugars in milk–tea [52]. The increase in total solids during concentration can cause the denaturation of whey proteins, leading to a more negative zeta potential (Table 2) due to the exposure of more charged groups. Prolonged heating may expose a large proportion of aspartic acid (Asp) and glutamic acid (Glu) residues, which are the main acid functional groups in whey proteins, leading to this effect [53]. Furthermore, the Maillard reaction, where lactose reacts readily, especially with water evaporation, leads to the formation of organic acids, resulting in a change in pH [18]. Additionally, an increase in solid concentration induces a collapse of casein micelles by forming swollen and diffuse micelles, which, at high concentrations, fragment into smaller structures [54]. This leads to a change in mineral balance. Further, it was found that β- and α_S_-casein slightly dissociate with concentration [55], which again destabilizes the micelles, resulting in changes in colloidal calcium phosphate (CCP). Similarly, it was reported that the concentration of ionic Ca^2+^ led to a significant reduction with increased concentration of milk, aligning well with the change in mineral balance resulting in a pH change [56]. The increase in micelle voluminosity also shifts soluble calcium and casein into the micelle, leading to a reduction in pH [54,57].

#### 3.1.2. Size

**Figure 2:**
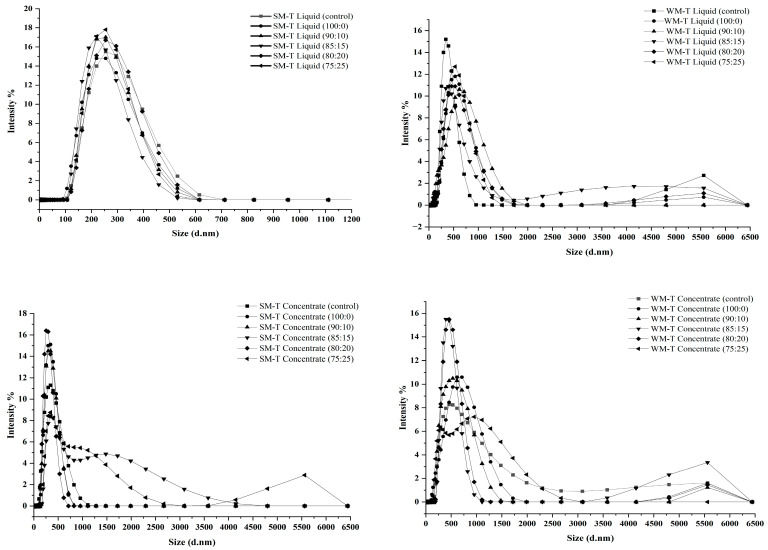
Particle size distribution profiles of SM-T and WM-T formulations across different L:M ratios, showing the effects of pasteurization and concentration (refer to Table S3).

The WM-T samples exhibited a larger z-average particle size compared to SM-T, both after pasteurization and concentration (Figure 2). This indicates the effect of the presence of fat globules, which can vary from 0.1 to 15 µm [35,58,59]. Concentration increased particle size for both WM-T, irrespective of the addition of lactose and maltodextrin, and SM-T. This is due to the increase in concentration of solutes leading to less intermolecular distances, thereby increasing the attraction forces, and hence, the additive effect of interactions between proteins themselves and fat globules during prolonged heating leads to aggregation. The reduction in water content increases the voluminosity of casein micelles due to the aggregation and interactions of whey proteins on the micelle surface [54]. However, Liu et al., (2012) [57] and Bienvenue et al. (2003) [60] reported that increased concentration decreases the size of micelles due to shrinking, which results from the saturation of CCP and dehydration of micelles. Interestingly, this decrease in size can be reversed at very high concentrations, where increased concentration promotes electrostatic repulsion between the particles. Cao et al., (2015) [61] reported that the increase in micelle voluminosity is mainly due to the attachment of whey proteins onto the micelle surface. Additionally, caseins contain a high amount of non-polar amino acids. Notably, β- and κ-caseins have numerous hydrophobic blocks in their sequences that can strongly bind to fatty acids [62,63]. β-casein has higher hydrophobicity compared to κ-casein and α-casein, involving hydrophobic amino acid residues such as proline (Pro) [64,65]. The presence of tea polyphenols further affects this aggregation. Polyphenols bind to both caseins and whey proteins through hydrophobic and hydrogen interactions. This binding can lead to the formation of larger aggregates or alter the size of existing casein micelles by forming complexes with the proteins.

WM-T liquid samples showed an increase in size with the addition of maltodextrin up to 10%, followed by a plateau with further additions up to 25%. In contrast, in concentrated WM-T samples, particle size decreased with increasing maltodextrin concentration up to 20%, followed by an increase with the addition of 25% maltodextrin. Maltodextrin can bind to milk proteins and polyphenols through hydrogen and hydrophobic bonding, stabilizing the protein structure and preventing further denaturation and aggregation. Increased maltodextrin concentration can encapsulate fat globules and prevent coalescence, leading to a stable size range. At higher concentrations, maltodextrin and maltodextrin–polyphenol complexes create a barrier around fat globules, which is more effective due to the higher density of maltodextrin molecules, thus preventing fat globules from merging into larger particles. Additionally, the high viscosity imparted by maltodextrin can reduce the mobility of fat globules and milk proteins, inhibiting their ability to collide and aggregate. Maltodextrin’s high affinity for water allows it to absorb significant amounts of moisture, leading to the hydration and swelling of maltodextrin. This, in turn, can encapsulate fat globules, polyphenols, and proteins more effectively. Interestingly, in both liquid and concentrated SM-T samples, the particle size remained almost unchanged with increasing maltodextrin addition, although a slight decrease was observed for concentrated samples, as seen in WM-T. In SM-T samples, the interaction between maltodextrin and proteins or polyphenols does not significantly alter particle size, suggesting that maltodextrin primarily acts to stabilize existing structures rather than forming new aggregates.

#### 3.1.3. BI and Total Phenolic Content

**Figure 3:**
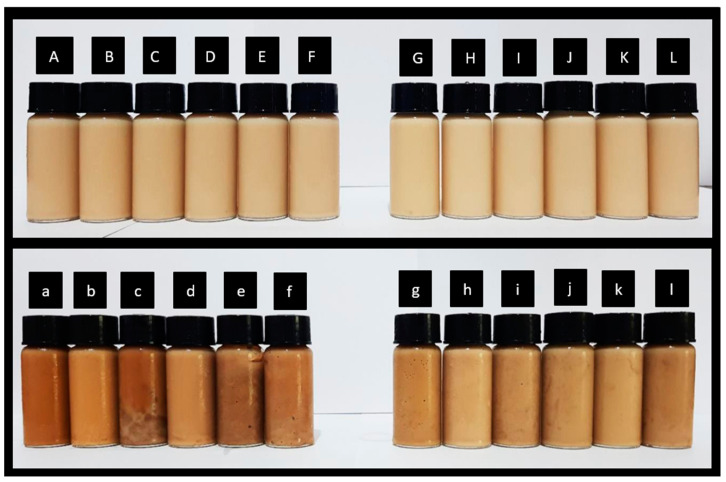
Different milk–tea liquid (A–L) and concentrated (a–l) formulations prepared with varying L:M (lactose/maltodextrin) ratios of 100:0, 90:10, 85:15, 80:20, 75:25. A–F: liquid-state skim milk–tea formulations (A—control; B—100:0; C—90:10; D—85:15; E—80:20; F—75:25); G–L: liquid-state whole milk–tea formulations (G—control; H—100:0; I—90:10; J—85:15; K—80:20; L—75:25); a–f: concentrated skim milk–tea formulations (a—control; b—100:0; c—90:10; d—85:15; e—80:20; f—75:25; g–l: concentrated whole milk–tea formulations (g—control; h—100:0; i—90:10; j—85:15; k—80:20; l—75:25).

Concentration increased the browning index (BI) irrespective of WM-T (*r*^2^ = 0.010, *p* < 0.05) or SM-T (*r*^2^ = −0.584, *p* < 0.05) (Table 1). For SM-T, the increase in BI with concentration decreased with the addition of maltodextrin (Table 2). This is attributed to the higher availability of lactose in the control sample, which participates more readily in the Maillard reaction. The rate of the Maillard reaction is related to the type of reactant in the following order: reducing monosaccharides > reducing polysaccharides > reducing 5-carbon sugars > reducing 6-carbon sugars [19]. Therefore, the high presence of lactose readily undergoes the Maillard reaction between the carbonyl group of lactose and the lysine residues of proteins, resulting in a higher brown color compared to samples with added maltodextrin (Figure 3) [66,67]. In contrast, the addition of maltodextrin increased the percentage increase in BI for WM-T. This may be due to the prevention of milk protein–maltodextrin complexation in the presence of fat, thereby providing more substrate for the Maillard reaction to occur.

The pasteurized WM-T control sample exhibited the highest total phenolic content of 969.47 ± 0.01 µg GAE/mL. Even after the variation in L:M ratios, WM-T had a higher phenolic content compared to SM-T samples. The highest TPC in WM-T samples as compared to SM-T can be attributed to the presence of naturally occurring fat-soluble phenolic compounds such as phenols, cresol, thymol, and carvacrol in milk [68]. Regardless of the L:M ratio, the TPC of both WM-T (*r*^2^ = −0.348, *p* < 0.05) and SM-T (*r*^2^ = −0.516, *p* < 0.05) decreased with the addition of maltodextrin after pasteurization, and this is due to interactions between phenolic compounds and maltodextrin. The hydroxyl group of polyphenols in tea and oxygen atoms of the glycosidic linkage of the polysaccharide produce hydrogen bonds and thereby result in the reduction in phenolic content after pasteurization [69]. 

Concentration leads to a further decrease in TPC in both WM-T and SM-T samples, as shown in Table 2. After concentration, the WM-T samples with 90:10 and 75:25 L:M ratios showed significantly higher retention of TPC compared to the control sample (*p* < 0.05). The reason for this is not entirely clear, but the combination of lactose and maltodextrin in specific ratios may lead to synergistic effects, enhancing their ability to preserve phenolic content after concentration. This interaction could result in a more effective stabilization mechanism. This preservation is achieved indirectly through various interactions, including electrostatic repulsion, whey–casein interactions, and Maillard reactions. Electrostatic repulsion plays a significant role in maintaining the stability of phenolic compounds in the WM-T samples. The electrostatic charges present on the surface of proteins and phenolic compounds can lead to repulsive forces, preventing their aggregation or degradation. Whey–casein interactions contribute to the preservation of phenolic compounds. The complexation between whey proteins, such as β-Lg, and casein proteins helps create a protective environment around the phenolic compounds, shielding them from degradation or interaction with other components in the blend. Moreover, Maillard reactions, which occur between amino groups of proteins and reducing sugars like lactose, can also indirectly contribute to the preservation of phenolic compounds. These reactions generate a variety of compounds, some of which may have antioxidant properties that aid in preserving phenolic compounds within the blend. These interactions collectively contribute to maintaining the stability and integrity of these bioactive compounds.

### 3.2. Structural Properties Affected by L:M Ratios of Liquid and Concentrated Milk–Tea 

#### 3.2.1. Polarized Micrographs 

**Figure 4:**
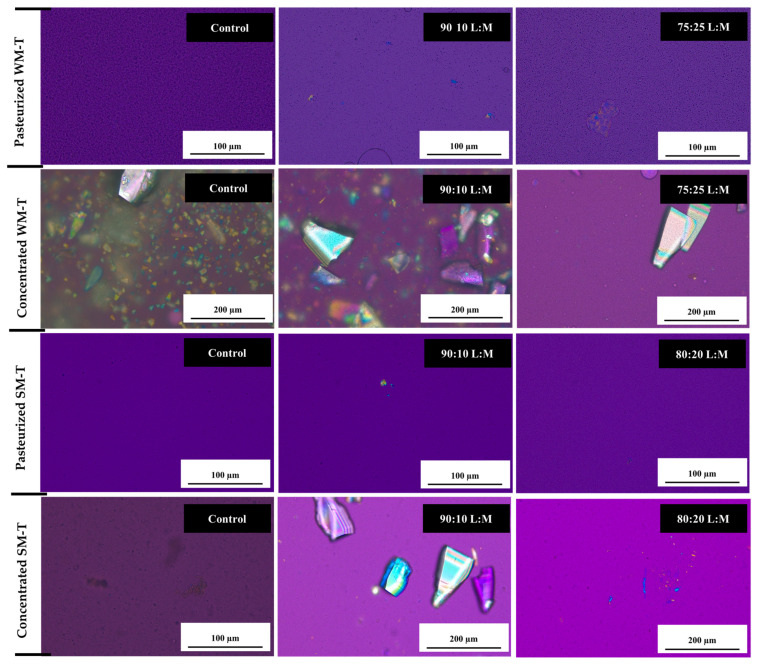
Polarized microscopy of pasteurized (liquid state) and concentrated whole and skim milk–tea formulations.

Initially, both SM-T and WM-T showed no lactose crystals, indicating that lactose remained in a non-crystalline, hydrated form as shown in Figure 4 [70]. However, during concentration, lactose concentration exceeded its solubility limit due to water removal, leading to supersaturation. This condition promoted nucleation, the initial step in crystal formation, where molecules aggregate into small, stable clusters. These clusters then serve as nuclei for further crystal growth. These clusters act as nuclei for further crystal growth, with additional lactose molecules attaching to them and resulting in larger crystals over time [71].

The addition of maltodextrin initially increased crystal growth in both SM-T and WM-T after concentration. However, higher maltodextrin concentrations resulted in fewer crystals, with a higher crystal count observed in WM-T compared to SM-T. This reduction in crystal formation with higher maltodextrin levels may be attributed to maltodextrin’s protective effect, as it forms hydrogen bonds with lactose and proteins, stabilizing the amorphous structure and reducing crystallization. Conversely, the presence of fat can interfere with lactose–protein interactions by promoting fat–protein interactions, which diminishes the stabilizing effect of maltodextrin and can lead to increased lactose crystallization [72,73]. 

#### 3.2.2. FTIR and PAGE Analysis

**Figure 5:**
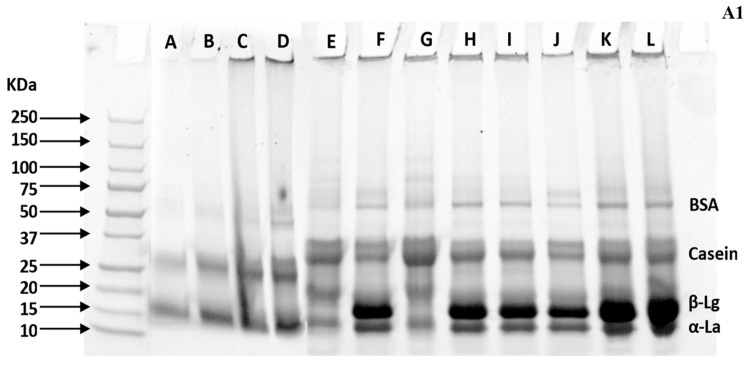

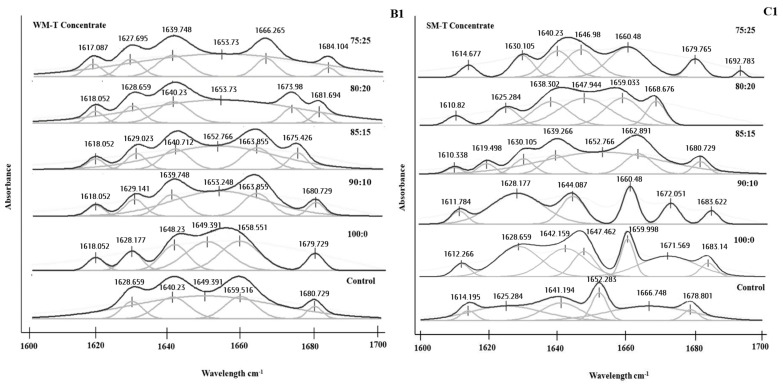
Visualization of individual protein fractions (**A1**) and secondary structure variation (**B1**,**C1**): non-reducing PAGE analysis of milk–tea formulation (**A1**): (A–C; WM-T liquid) A: control, B: 90:10, C: 75–25; (D–F; WM-T concentrate) D: control, E: 90:10, F: 75–25; (G–I; SM-T liquid) **G**: control, H: 90:10, I: 80:20; (J–L; SM-T concentrate) J: control, K: 90:10, **L**: 80:20. Secondary structure changes of protein (FTIR) in (**B1**) WM-T and (**C1**) SM-T.

Initially, the SM-T formulation exhibited significantly higher band intensity for κ-casein compared to the WM-T formulation as shown in Figure 5, both after pasteurization and concentration, due to the higher proportion of κ-casein in skim milk. In contrast, the WM-T formulation displayed a more balanced distribution of α-La, β-Lg, and κ-casein, as detailed in Table 3. During concentration, the β-Lg concentration in WM-T decreased, while κ-casein substantially increased due to the formation of β-Lg–κ-casein complexes. At approximately pH 7, β-Lg binds to both free and surface-active κ-casein, a process facilitated by prolonged heating and reduced particle distance, which promotes the association of casein micelles [74,75,76]. Additionally, polyphenols such as flavonoids and epigallocatechin gallate interact with β-Lg, forming hydrophobic and hydrogen bonding interactions that further reduce β-Lg levels. The presence of fatty acids, such as palmitic, oleic, and myristic acids in whole milk, also encourages β-Lg reduction at pH 7 and temperatures below its denaturation point due to interactions between fatty acids and protein [77].

Pasteurization induced conformational changes in proteins in both SM-T and WM-T formulations with the addition of lactose and maltodextrin. Increased addition of maltodextrin led to a decrease in α-La and β-Lg while increasing κ-casein. For instance, the addition of 25% maltodextrin reduced α-La and β-Lg by 25% and 14%, respectively, while increasing κ-casein by 26%. This change can be attributed to protein–protein interactions, which may include β-Lg–β-Lg, β-Lg–α-La, β-Lg–κ-casein, and/or α-La–κ-casein interactions. The increased maltodextrin provides a higher number of hydroxyl groups, forming hydrogen bonds with the hydrophilic regions of proteins. This bonding causes the unfolding of protein structures, exposing hydrophobic regions. These hydrophobic regions can result in inter- or intra-protein interactions, reducing the amounts of β-Lg and α-La. β-Lg–κ-casein and α-La–κ-casein complexes might be the predominant protein–protein interactions, as indicated by the increase in κ-casein along with a slight reduction in β-Lg and α-La. The presence of fat further encourages these hydrophobic interactions, reducing β-Lg and α-La compared to SM-T [78]. In contrast, SM-T showed an increase in α-La and β-Lg by 51% and 3%, respectively, while κ-casein decreased by 8% with the addition of 25% maltodextrin. This is due to the lower fat content in SM-T, which reduces the hydrophobic regions available to stabilize the protein structure. 

The concentration process resulted in a significant reduction in α-La and κ-casein in WM-T, while β-Lg levels increased with the addition of maltodextrin. FTIR analysis supports this observation (Table 4), showing changes in protein structure. α-La predominantly has an α-helix configuration, comprising about 47% of its structure with roughly 6% β-sheet content, whereas β-Lg has 15% α-helix and 50% β-sheet content [79]. The rise in β-sheet structures at 1610–1642 cm⁻^1^ and 1688–1697 cm⁻^1^ confirms the increase in β-Lg levels in WM-T. The increase in α-La and β-Lg levels, alongside the reduction in κ-casein after concentration with added maltodextrin, indicates that protein structures refold and stabilize due to decreased protein–protein and protein–lactose interactions. Maltodextrin acts as a crowding agent, promoting protein stability by reducing protein–protein interactions through the excluded volume effect, where maltodextrin molecules occupy space and limit the available volume for protein molecules, leading to a reduced likelihood of aggregation [80]. Decreased protein–lactose interactions are due to a reduced Maillard reaction, which occurs because polysaccharides have fewer carbonyl groups. This reduction in the Maillard reaction helps reduce protein conformational changes and retain more lysine residues in the protein [81]. This is supported by research which shows that increasing the amino/carbonyl ratio from 1:1 to 1:2 increased glycation by 3.5%, and when the ratio increased from 1:3 to 1:15 in conjugates formed between bovine serum albumin and galactomannan, it resulted in the binding of 2.5 to 6.7 molecules of polysaccharide to the protein [82]. Further, this stability is confirmed by FTIR results, which show the absence of random coils at 1644–1648 cm⁻^1^ and large loops at 1656–1658 cm⁻^1^ after concentration with maltodextrin addition, indicating that these flexible structures have transformed into more stable forms such as α-helices, β-sheets, and β-turns upon concentration. In contrast, SM-T exhibited an increase in large loops at 1659 cm⁻^1^ and random coils at 1644 cm⁻^1^ and 1648 cm⁻^1^, indicating that the absence of fat did not support the same level of protein stability as observed in WM-T. This reduced stability in SM-T is confirmed by PAGE results, which show an increase in κ-casein with higher maltodextrin levels. This suggests that the absence of fat affects the protein stabilization properties of maltodextrin.

## 4. Mechanisms for the Preservation Effect of Polyphenols

**Figure 6:**
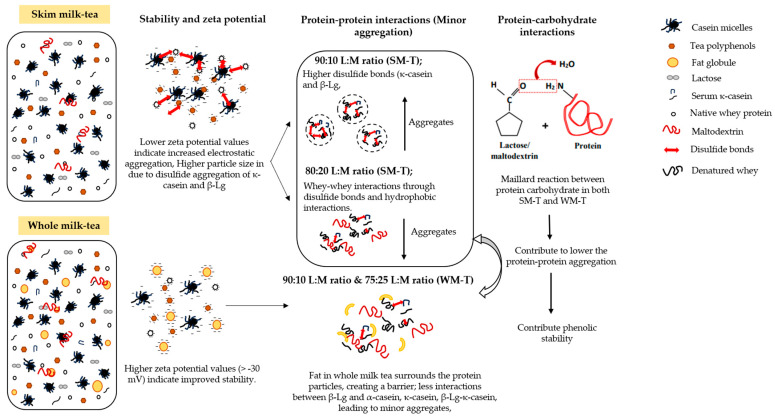
Mechanism of stabilizing tea polyphenols in milk–tea with varying L:M ratios.

Different ratios of maltodextrin stabilize the protein structure and polyphenol structure by various interaction mechanisms as shown in Figure 6. Milk–tea formulations with 90:10 and 75:25 L:M ratios exhibited a significantly pronounced TPC protection effect in the presence of fat and maltodextrin compared to formulations without these components.

The protective effect of polyphenol structures can be explained by several mechanisms involving pH stabilization, zeta potential modulation, protein interactions, complex formation, and encapsulation effects: (1) Adding maltodextrin and lactose to milk–tea samples dilutes acidic components, buffering H^+^ ions and raising pH. This pH increase during pasteurization reduces overall acidity. Polyphenols, sensitive to pH, benefit from this higher pH as it minimizes their degradation, typically accelerated by acidic conditions. Additionally, polyphenol–protein interactions and maltodextrin–fat cross-linking stabilize pH by sequestering H^+^ ions, further reducing polyphenol degradation. (2) Elevated zeta potential values in WM-T and SM-T samples indicate strong negative charges on protein-rich surfaces, enhanced by tea polyphenols. Higher maltodextrin concentrations increase the zeta potential, enhancing electrostatic stabilization and preventing polyphenol and protein aggregation, thereby maintaining polyphenolic stability. (3) Further, maltodextrin binds to polyphenols through hydrogen bonding and hydrophobic interactions, shielding them from oxidation and improving solubility. It also interacts with proteins via hydroxyl groups, altering protein conformation to prevent denaturation and create a favorable environment for polyphenols. (4) Further, maltodextrin and polyphenols form complexes with milk proteins such as κ-casein, β-Lg, and α-La, acting as protective barriers against oxidative stress and enzymatic degradation. These complexes encapsulate polyphenols, embedding them within a matrix of proteins and polysaccharides, thus maintaining phenolic stability under processing conditions. (5) Maltodextrin’s high water-absorbing capacity enhances hydration and encapsulates polyphenols and fat globules, reducing their mobility and interaction with degrading agents. Increased viscosity and hydration prevent fat globule coalescence and protect polyphenols. (6) Maltodextrin moderates the Maillard reaction by interacting with amino acids and reducing sugars like lactose, thereby preventing excessive browning and preserving phenolic compounds that might otherwise degrade. (7) Additionally, the Maillard reaction promotes only minimal protein–protein aggregation, which enhances emulsion stability. By reducing excessive protein–protein interactions, the Maillard reaction contributes to a more stable emulsion in milk–tea formulations. This stability helps maintain a consistent environment, which reduces the exposure of phenolic compounds to oxidative conditions and thereby protects them from degradation. As a result, the preservation of phenolic compounds is improved, leading to enhanced quality and nutritional value of the final product.

However, the absence of fat does not pronounce this preservation effect of polyphenols compared to WM-T due to a less balanced distribution of nutrients. Specific L:M ratios in SM-T, such as 90:10 and 80:20, showed an enhanced polyphenol preservation effect while 90:10 and 75:25 showed enhanced TPC. This is exhibited by the systematic representation in Figure 6, which shows how these mechanisms interplay differently with composition variations in WM-T and SM-T with specific L:M ratios. 

In practical terms, preserving phenolic compounds in milk–tea formulations enhances their bioavailability and mitigates the reduction in bioavailability typically observed with tea due to factors such as oxidation or interaction with other ingredients. This preservation is crucial because phenolic compounds are associated with significant health benefits, including chemo-preventive activity against various cancers, antioxidant effects that combat oxidative damage, a lowering effect on coronary heart disease, and protection against dental caries and bone loss. By modifying the L:M ratio, one can effectively control the phenolic masking effect, thereby significantly impacting the health benefits of these compounds. For instance, increasing the maltodextrin content relative to lactose can stabilize phenolic compounds, preventing their degradation and loss of biological activity during processing [83,84].

Consequently, optimizing the L:M ratio not only improves the preservation of phenolics but also enhances their functional benefits, such as antioxidant activity, compared to formulations using natural milk compositions. This approach ensures that the health-promoting properties of phenolic compounds are maximized, leading to a more beneficial product.

## 5. Conclusions

This study investigated the effects of different L:M ratios on the physical and structural properties of pasteurized and concentrated skim and whole milk–tea blends. The key findings demonstrate that varying L:M ratios significantly influence the protein and phenolic interactions within milk–tea formulations. The results reveal that increasing the maltodextrin content relative to lactose enhances the stability of casein micelles and whey proteins, which in turn affects the stability of the milk–tea blends. Notably, higher maltodextrin concentrations contribute to improved phenolic retention, especially in fat-filled milk–tea, by forming protective complexes that shield phenolic compounds from thermal degradation. Several mechanisms contribute to this effect: Electrostatic repulsion among negatively charged proteins, carbohydrates, polyphenols, and fats effectively prevents polyphenol aggregation. Additionally, Maillard reaction-derived compounds may indirectly support phenolic preservation by interacting with low-molecular-weight Maillard products. Furthermore, structural changes in proteins create a protective matrix for phenolics. In WM-T, minor disulfide aggregation of κ-casein with β-Lg and α-La contributes to polyphenol stabilization, while in SM-T, both disulfide aggregation and hydrophobic interactions among whey proteins play a role. The beneficial effects of specific L:M ratios were observed in blends with ratios of 90:10 and 75:25 in WM-T and 90:10 and 80:20 in SM-T. These ratios demonstrated superior phenolic preservation after concentration and suggest that optimizing L:M ratios may enhance the properties of milk–tea blends more effectively than using milk with its natural composition. 

## Figures and Tables

**Table 1:** The Pearson correlation coefficients between L:M ratio and zeta potential, total phenolic content (TPC), and browning index (BI) after pasteurization and concentration.

WM-T	Liquid			Concentrate		
	Zeta Potential	TPC	BI	Zeta Potential	TPC	BI
L:M Ratio	0.252	−0.348	−0.099	0.091	0.462	0.010
SM-T	Liquid			Concentrate		
L:M Ratio	0.648 **	−0.516 *	−0.931 **	0.084	−0.368	−0.584 *

** Correlation is significant at the 0.01 level (2-tailed). * Correlation is significant at the 0.05 level (2-tailed).

**Table 2:** Zeta potential, TPC, and BI change in SM-T and WM-T after pasteurization and concentration.

L:M Ratio	Physiochemical Property (SM-T Liquid)			Physiochemical Property (SM-T Concentrate)		
	Zeta Potential (mV)	TPC	BI	Zeta Potential (mV)	TPC	BI
		(µg GAE/mL)			(µg GAE/mL)	
Control	−29.30 ± 1.15 ^Bb^	792.28 ± 3.04 ^d^	42.64 ± 0.19 ^Eb^	−28.93 ± 0.84 ^Bd^	649.59 ± 1.02 ^e^	77.80 ± 19.30 ^Ca^
100:0	−29.28 ± 0.88 ^Bb^	701.05 ± 1.76 ^c^	40.30 ± 1.52 ^Dc^	−31.06 ± 2.12 ^Ac^	564.79 ± 1.01 ^c^	73.17 ± 1.07 ^Ba^
90:10	−29.22 ± 1.01 ^Bb^	605.15 ± 1.02 ^a^	35.47 ± 1.93 ^Ba^	−28.78 ± 2.13 ^Bd^	567.13 ± 4.05 ^c^	64.63 ± 1.21 ^Ba^
85:15	−27.17 ± 0.45 ^Cc^	680.00 ± 4.64 ^b^	36.69 ± 0.51 ^Ca^	−30.24 ± 1.27 ^Bc^	425.03 ± 2.68 ^a^	61.95 ± 11.1 ^Ba^
80:20	−27.60 ± 0.44 ^Bc^	681.75 ± 3.51 ^b^	35.59 ± 0.57 ^Ba^	−30.13 ± 0.58 ^Bc^	622.11 ± 1.76 ^d^	54.31 ± 8.26 ^Aa^
75:25	−27.25 ± 1.83 ^Cc^	671.82 ± 8.29 ^b^	43.81 ± 0.79 ^Fd^	−28.69 ± 0.81 ^Bd^	535.56 ± 3.65 ^b^	62.87 ± 5.19 ^Ba^
**L:M Ratio**	**Physiochemical Property (WM-T Liquid)**			**Physiochemical Property (WM-T Concentrate)**		
	**Zeta Potential (mV)**	**TPC**	**BI**	**Zeta Potential (mV)**	**TPC**	**BI**
		**(µg GAE/mL)**			**(µg GAE/mL)**	
Control	−30.52 ± 0.88 ^Cb^	969.47 ± 0.01 ^e^	44.37 ± 0.19 ^Fe^	−33.81 ± 0.82 ^Ba^	677.08 ± 1.02 ^b^	67.45 ± 4.32 ^Ba^
100:0	−29.99 ± 0.36 ^Db^	857.78 ± 9.97 ^c^	44.60 ± 0.89 ^Fe^	−33.41 ± 0.89 ^Ba^	639.06 ± 9.00 ^a^	70.74 ± 2.40 ^Ba^
90:10	−31.66 ± 1.35 ^Ca^	809.83 ± 9.77 ^b^	43.16 ± 0.39 ^Ed^	−31.99 ± 0.83 ^Cc^	709.82 ± 5.27 ^c^	63.89 ± 3.17 ^Ba^
85:15	−31.65 ± 1.00 ^Ca^	922.11 ± 7.65 ^d^	40.44 ± 2.28 ^Dc^	−34.21 ± 1.70 ^Aa^	696.96 ± 5.64 ^c^	66.74 ± 5.03 ^Ba^
80:20	−30.17 ± 2.02 ^Db^	759.53 ± 2.68 ^a^	36.61 ± 0.73 ^Cb^	−32.88 ± 1.70 ^Bb^	651.34 ± 2.68 ^a^	63.60 ± 4.80 ^Ba^
75:25	−28.97 ± 1.31 ^Eb^	905.15 ± 1.02 ^d^	32.65 ± 0.36 ^Aa^	−33.19 ± 1.75 ^Ba^	736.14 ± 6.32 ^d^	71.36 ± 5.96 ^Ba^

For BI and TPC: lowercase superscripts within each type of milk–tea (SM-T or WM-T) in the same physical state (liquid or concentrate) indicate significant differences (*p* < 0.05); uppercase superscripts between SM-T and WM-T within the same physical state indicate significant differences (*p* < 0.05). For zeta potential: different lowercase superscripts between SM-T and WM-T within the same physical state (liquid or concentrate) indicate significant differences (*p* < 0.05); different uppercase superscripts within SM-T or WM-T in different physical states (liquid or concentrate) indicate significant differences (*p* < 0.05).

**Table 3:** Changes in individual protein fractions with varying L:M ratios in formulations after pasteurization and concentration (refer to Figure 5A1).

L:M Ratio	WM-T Liquid %			WM-T Concentrate %		
	α-La	β-Lg	κ-Casein	α-La	β-Lg	κ-Casein
Control	16.19	20.23	32.14	27.52	8.43	51.43
90:10	14.77	19.22	32.69	16.70	25.37	36.08
75:25	12.13	17.38	40.45	25.65	25.51	35.92
	**SM-T Liquid %**			**SM-T Concentrate %**		
Control	8.43	48.35	76.79	27.80	51.52	73.07
90:10	12.65	18.16	99.91	24.69	46.01	72.10
80:20	12.71	49.64	70.91	22.49	40.56	75.93

**Table 4:** Secondary structure changes of protein with different L:M ratios during concentration (refer to Figure 5; B1 & C1).

Secondary Structure	SM-T Concentrate (%)						WM-T Concentrate (%)					
	Control	100:0	90:10	85:15	80:20	75:25	Control	100:0	90:10	85:15	80:20	75:25
β-sheets	55.76	52.18	50.46	24.09	34.29	38.57	20.43	32.60	24.89	22.38	23.03	21.62
Random coil	0.00	11.03	18.56	0.00	32.26	23.14	62.91	31.15	0.00	0.00	0.00	0.00
α-helices	11.74	0.00	0.00	59.04	0.00	0.00	0.00	0.00	55.31	57.59	68.72	69.45
Large loops	0.00	9.93	0.00	0.00	23.04	0.00	13.10	28.95	0.00	0.00	0.00	0.00
β-turns	32.50	26.86	30.99	16.87	10.40	38.29	3.57	7.30	19.80	20.03	8.25	8.92

## Data Availability

The original contributions presented in the study are included in the article/Supplementary Materials, further inquiries can be directed to the corresponding author.

## Supplementary Materials

The following supporting information can be downloaded at: https://www.mdpi.com/article/10.3390/foods13183016/s1, Table S1: Composition of ten different milk-tea formulas (SM-P containing 54.2% lactose, 35.4% proteins, 1.30% fat and 9.11% minerals, WM-P containing 41.59% lactose, 25.28% protein, 27.14% fat, 5.99% minerals) (SM-P = Skim milk powder, WM-P=Whole milk powder); Table S2: pH changes of L:M ratio varied (A)WM-T & (B) SM-T during mixing, adjusting pH, pasteurization, homogenization and concentration (Refer to Figure 1); Table S3: Z-Average particle size (in nm) of milk-tea formulations with varying L:M ratios, measured in both liquid and concentrate forms (Refer to Figure 2).
