# Supplementary material for: The Impact of Varying Lactose-to-Maltodextrin Ratios on the Physicochemical and Structural Characteristics of Pasteurized and Concentrated Skim and Whole Milk–Tea Blends
Source: Foods. 2024 Sep 23;13(18):3016. doi: 10.3390/foods13183016 (PMC11431367; doi:10.3390/foods13183016)
Supplement: Supplementary file 1 [file foods-13-03016-s001.zip › foods-3172874-supplementary.pdf]

Supplementary Materials (S)

Table S1. composition of ten different milk-tea formulas (SM-P containing 54.2% lactose, 35.4% proteins, 1.30% fat and 9.11% minerals, WM-P containing 41.59% lactose, 25.28% protein, 27.14% fat, 5.99% minerals) (SM-P=Skim milk powder, WM-P=Whole milk powder)

L:M ratio	Lactose	Maltodextrin	SM-P	Tea concentration %	Final formulation (milk+tea)
100:0	19.68	0.00	90.0	2	546.9
90:10	12.74	6.81	90.0	2	546.9
85:15	9.26	10.21	90.0	2	546.9
80:20	5.79	13.62	90.0	2	546.9
75:15	2.32	17.03	90.0	2	546.9
L:M ratio	Lactose	Maltodextrin	WM-P	Tea concentration %	Water
100:0	15.76	0.00	90.0	2	527.0
90:10	10.37	5.28	90.0	2	527.0
85:15	7.68	7.92	90.0	2	527.0
80:20	4.99	10.56	90.0	2	527.0
75:25	2.29	13.20	90.0	2	527.0

Table S2. pH changes of L:M ratio varied (A) WM-T & (B) SM-T during mixing, adjusting pH, pasteurization, homogenization and concentration (Refer to figure 1).

**L:M ratio**	**WM-T**				**SM-T**			
	**After mixing**	**After pasteurization**	**After homogenizing**	**After concentration**	**After mixing**	**After pasteurization**	**After homogenizing**	**After concentration**
Control	6.55±0.04^{a}	7.38±0.15^{d}	7.14±0.04^{e}	7.11±0.01^{e}	6.65±0.03^{a}	6.91±0.02^{b}	7.16±0.06^{c}	6.72±0.02^{b}
100:0	6.74±0.03^{e}	6.85±0.04^{b}	6.86±0.04^{b}	6.64±0.03^{b}	6.73±0.02^{b}	6.82±0.02^{a}	6.84±0.03^{a}	6.56±0.03^{a}
90:10	6.64±0.04^{c}	7.03±0.06^{c}	6.95±0.03^{d}	6.75±0.04^{c}	6.74±0.04^{b}	7.09±0.01^{c}	6.95±0.04^{b}	6.76±0.03^{d}
85:15	6.72±0.03^{d}	6.95±0.05^{b}	6.93±0.02^{c}	6.74±0.03^{c}	6.75±0.01^{b}	6.82±0.03^{a}	6.83±0.02^{a}	6.80±0.01^{e}
80:20	6.76±0.03^{e}	6.82±0.02^{a}	6.83±0.01^{a}	6.56±0.02^{a}	6.73±0.02^{b}	6.93±0.03^{b}	6.95±0.04^{b}	6.83±0.01^{f}
75:25	6.61±0.01^{b}	6.95±0.04^{b}	6.94±0.02^{d}	6.92±0.01^{d}	6.75±0.02^{b}	6.96±0.02^{b}	6.95±0.02^{b}	6.73±0.04^{c}

Lowercase superscripts within each processing stage (mixing, pasteurization, homogenizing, and concentration) for the same milk-tea formulation indicate significant differences ($p < 0.05$)

Table S3. Z-Average particle size (in nm) of milk-tea formulations with varying L:M ratios, measured in both liquid and concentrate forms (Refer to Figure 2).

L:M ratio	Z-Average particle size (d.nm) of SM-T		Z-Average particle size (d.nm) of WM-T	
	Liquid	Concentrate	Liquid	Concentrate
Control	240.5±97.39[a]	286.7±183.1[a]	407.2±132.5[a]	549.7 ±134.1[a]
100:0	214.5±90.63[b]	277.7±153.5[b]	445.3±249.8[a]	533.9±312.2[a]
90:10	237.8±82.09[a]	298.8±162.9[b]	457.0±245.0[a]	516.5±249.4[a]
85:15	225.4±75.08[b]	282.7±182.4[b]	439.5±249.5[a]	480.2±146.4[a]
80:20	248.5±87.91[a]	275.1±132.1[b]	447.5±265.7[a]	457.2±165.7[a]
75:25	234.9±78.07[a]	331.5±114.6[b]	438.7±221.9[a]	484.1±256.7[a]

Lowercase superscripts within each physical state (liquid or concentrate) for the same milk-tea formulation indicate significant differences ($p < 0.05$)
